# Novel anti-inflammatory role of SLPI in adipose tissue and its regulation by high fat diet

**DOI:** 10.1186/1476-9255-8-5

**Published:** 2011-02-28

**Authors:** Venkata J Adapala, Kimberly K Buhman, Kolapo M Ajuwon

**Affiliations:** 1 Department of Animal Sciences, Purdue University, West Lafayette, Indiana, 47907, USA; 2 Department of Foods and Nutrition, Purdue University, West Lafayette, Indiana, 47907, USA

## Abstract

**Background:**

Secretory leucocyte protease inhibitor (SLPI) is an anti-inflammatory protein that is constitutively expressed in multiple cell types where it functions to counteract localized tissue inflammation by its anti-inflammatory, antimicrobial and anti-protease properties. Little is known about the expression and implication of SLPI in the regulation of adipose tissue inflammation. Therefore, we tested the hypothesis that obesity induces expression of SLPI in adipose tissue where it functions to counteract adipocyte inflammation.

**Methods:**

Male C57BL6 mice were fed a high fat (60% fat calories) or a control diet (10% fat calories) diet for 12 weeks. Adipose tissue expression of SLPI was determined by western blotting and PCR. Fully differentiated adipocytes (3T3-L1) were treated with lipopolysaccharide (LPS, 100 ng/ml) or peptidoglycan (10 μg/ml) for 24 hours in the presence or absence of SLPI. Media was collected for interleukin 6 (IL-6) analysis by enzyme-linked immune absorbent assay (ELISA). RNA was isolated for gene expression analysis by real-time polymerase chain reaction (RT-PCR).

**Results:**

Visceral fat (mesenteric and epididymal) express a higher level of SLPI than subcutaneous fat. The expression of SLPI is mostly in the stromal vascular fraction compared to adipocytes. We also confirmed in vitro that activation of TLR2 and 4 with peptidoglycan and LPS respectively leads to induction of SLPI. Finally, we confirmed that SLPI exerted an anti-inflammatory effect in adipocytes treated with LPS by causing a reduction in expression of IL-6 via a mechanism that included stabilization of cellular IKBα expression.

**Conclusion:**

Our results show that SLPI is also expressed in adipocytes and adipose tissue where it could play an important feedback role in the resolution of inflammation.

## Background

Obesity is associated with adipose tissue inflammation that eventually results in insulin resistance. This is characterized by adipose tissue macrophage infiltration [1,2], elevated expression of inflammatory cytokines, including TNFα [3], IL6 [4], monocyte chemoattractant protein (MCP) 1 [5], plasminogen activator inhibitor (PAI) 1[6]. Inflammatory cytokines produced in adipose tissue act locally and systemically to amplify the inflammatory cascade and oppose insulin signaling in peripheral tissues. However, little is known about mechanisms that lead to resolution of inflammation in adipose tissue. Secretory leucocyte protease inhibitor (SLPI) is a protein that may play a major role in the dampening of inflammation in adipose tissue. It is an 11.7-kD non-glycosylated protein produced primarily at mucosal surfaces, especially in the upper respiratory tract [7]. In the lung [8], SLPI interacts and inhibits the activity of several proteolytic enzymes, making it an integral component of the defense mechanism in the lung. Apart from its anti-protease activity, SLPI also exerts anti-inflammatory effect against viral and antibacterial targets [9]. SLPI also inhibits NF-κB activation and production of TNF-α and nitric oxide [10] and SLPI knockout mice have an exaggerated inflammatory response and go into septic shock after LPS administration [11]. Although SLPI is expressed at multiple tissues during inflammation where it acts to counter the inflammatory events, there is no report of adipose tissue expression of SLPI or a potential anti-inflammatory role of SLPI in adipocytes. Therefore, we examined its expression in adipose tissue of mice that have been fed a high fat diet and in 3T3-L1 adipocytes treated with ligands for both toll-like receptors (TLR) 2 and 4, two major inflammatory receptors in adipose tissue [12,13 ].

We demonstrate herein, for the first time, that SLPI is upregulated in adipose tissue in obesity. Additionally, we show that SLPI opposes induction of IL6 by LPS in adipocytes. Therefore, SLPI could be a potential target for the regulation of inflammation in adipose tissue.

## Methods

### 3T3-L1 Adipocyte Culture

Cells were obtained from ATCC (Manassas, VA) and cultured according to standard conditions. Briefly, cells were grown under 5% CO2 in Dulbecco's Modified Eagles Medium (DMEM) containing 10% fetal bovine serum (Hyclone, Logan, UT) and 0.5% penicillin-streptomycin mixture (Invitrogen, Carlsbad, CA). Cells were allowed to reach confluence, and two days post confluence (day 0), were induced to differentiate with a medium containing 10% fetal bovine serum, 1.7 μM insulin, 1 μM dexamethasone, and 0.5 mM IBMX for 48 h. Thereafter, fresh medium containing only insulin and fetal bovine serum was added for another 2 days. From then on media was replenished every 2 days with DMEM containing only 10% FBS. Fully differentiated cells were treated for 24 hours with either *Staphylococcus aureus *derived peptidoglycan (10 μg/mL) or *E.coli *lipopolysaccharride (100 ng/mL) (Sigma, St. Louis. MO).

### Animals

Eight week old male C57BL/6J mice were fed either a high fat diet (HF, D12492i) with 60% fat calories (n = 8) or a control diet (LF, D12450Bi) with 10% calories (n = 8) from fat (Research Diets, New Brunswick, NJ, http://www.researchdiets.com) for 12 weeks. At the end of the experiment animals were euthanized by CO_2 _asphyxiation followed by cervical dislocation. All animal care protocols were approved by the Purdue Animal Care and Use Committee. Epididymal adipose tissue was obtained by careful dissection of adipose tissue around the epididymis and used for RNA extraction with Trizol (Invitrogen, Carlsbad, CA) or tissue lysates for western blotting. We also collected subcutaneous (collected from underneath the skin around the lumbar area), mesenteric (collected by careful dissection of adipose tissue from around the intestine) for a comparative analysis of SLPI mRNA expression by real-time PCR. To determine the relative expression of SLPI in adipocytes and stromal vascular fraction (SVF), adipose tissue was subjected to collagenase digestion (1 mg/ml Collagenase type 1, Sigma) in Krebs Ringer Buffer (118.5 mM NaCl, 4.8 mM KCl, 2.7 mM CaCl_2_, 1.2 mM KH_2_PO_4_, 1.1 mM MgSO_4_, 7H _2_O, 25 mM NaHCO_3_, 5 mM glucose and 5% (w/v) BSA, pH 7.4) with shaking at 150 RPM for 30 minutes at 37°C. After digestion, adipocytes were allowed to separate by flotation and the infranatant solution was centrifuged for 5 minutes at 300 g to pellet the stromovascular fraction (SVF). The adipocyte fraction was washed three times with the KRB buffer to remove contaminants and ensure a pure population of adipocytes. This method has been validated with flow cytometry to yield a 100% pure population of adipocytes. Subsequently, RNA was isolated from adipocytes and the SVF for comparison with whole adipose tissue.

### Anti-inflammatory effect of SLPI

Differentiated 3T3-L1 adipocytes were pretreated for 2 hours with 10 ng/ml recombinant human SLPI (R &D Systems, Minneapolis, MN) and then treated with LPS for 3 hours. Media was recovered for ELISA and RNA for RT-PCR.

### Real-time quantitative RT-PCR

Total RNA from treated cells was extracted with Trizol Reagent (Invitrogen) according to the manufacturer's protocol. The mRNAs were treated with Turbo DNase (Ambion, Austin, TX) to remove contaminating DNA and reverse transcribed into cDNA using Improm II reverse transcriptase (Promega, Madison, WI). Real-time PCR was performed using a MyIQ real-time PCR detection machine (Bio-Rad) with the Faststart SYBR green based mix (Roche, Indianapolis, IN). Primers sequences used were: IL-6, 5'-AACGATGATGCACTTGCAGA-3' and 5'-GAGCATTGGAAATTGGGGTA-3' for the sense and antisense primers, respectively (14); SLPI, sense, 5'-TGCTTAACCCTCCCAATGTC-3' and antisense, 5'-AATGCTGAGCCAAAAGGAGA-3'; β-actin sense, 5'-ATGGGTCAGAAGGACTCCTACG-3' and antisense, 5'-AGTGGTACGACCAGAGGCATAC-3'; TNFα, 5'-AGCCCCCAGTCTGTATCCTT-3' and 5'-CTCCCTTTGCAGAACTCAGG-3'. Quantification of transcripts was done with the ΔΔ Ct method with normalization against the β-actin.

### Immunoblotting

Whole tissue lysates were obtained by homogenizing tissues and cells in RIPA lysis buffer (0.5 M Tris-HCl, 1.5 M HCl, 2.5% Deoxycholic acid, 10% NP-40 and 10 mM EDTA) supplemented with protease and phosphates inhibitor cocktail (Sigma). Homogenized tissues and cells were then cleared of cellular and tissue debris by centrifugation at 10,000 g for 10 minutes at 4°C. Protein concentrations were determined with the BCA kit (Sigma). For immunoblotting, 50 μg of lysates were resolved on a 10% SDS-PAGE gel and transferred to a nitrocellulose membrane. Membranes were probed with rabbit anti-SLPI (Cat # SC-28803, Santa Cruz, CA, USA) primary antibody and HRP-conjugated anti-rabbit secondary antibody (Cat# 7074, Cell Signaling, Danvers, MA, USA). To determine the role of IKBα protein in the regulation of SLPI effect, the expression of phosphorylated and native IKBα was quantified by western blotting using rabbit primary antibodies (Cat# 2859 and 4812, Cell Signaling, Danvers, MA, USA). Blots were subsequently blotted with the Supersignal^® ^West Pico chemilumniscent reagent (Pierce, Rockford, IL) and exposed to autoradiographic film to capture protein specific signals.

### ELISA for Media IL-6

Media concentration of IL-6 was determined with a mouse IL-6 ELISA kit (Endogen, Rockford, IL) according to the manufacturer's instructions. This kit has an assay sensitivity of < 7 pg/ml and an inter assay and intra assay variation of < 10%.

### Statistical analyses

All data were checked for normality and then analyzed using the GLM model analysis. When treatment effects were significant, mean separation was accomplished using the least-squares mean separation procedure.

## Results

### Adipose tissue expression of SLPI and regulation by high fat diet

**Figure 1:**
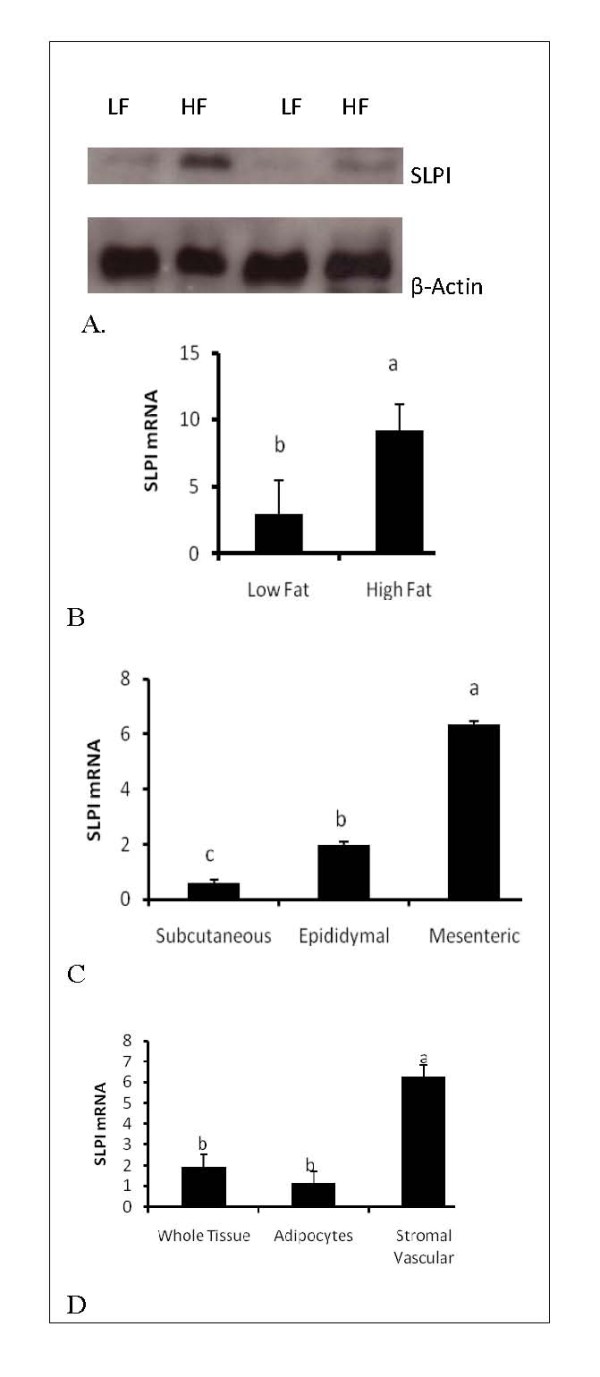
**High fat feeding increases SLPI expression in adipose tissue of mice**. Mice were fed either a control low fat (LF) or high fat (HF) diet for 12 weeks. Epididymal adipose tissue were obtained and subjected to western blotting for SLPI protein. A representative blot is presented in Figure 1A. Expression of SLPI mRNA was quantified in Figures 1B, 1C and 1D. High fat feeding increases SLPI mRNA (Figure 1B) in adipose tissue. Higher expression of SLPI was observed in epididymal and mesenteric depots compared to the subcutaneous depot (Figure 1C), and in the stromal vascular faction compared to adipocytes (Figure 1D). Bars represent means and ± SEM. Superscript letters represent significant mean differences, P < 0.05.

**Figure 2:**
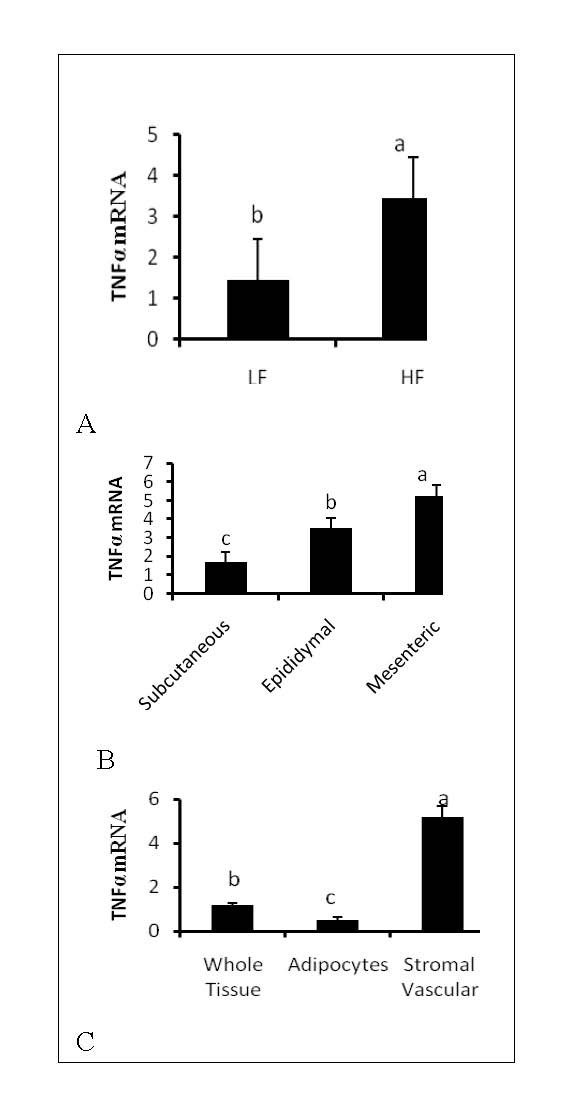
**High fat feeding increases Inflammation in adipose tissue of mice**. Mice were fed either a low fat (LF) or high fat (HF) diet for 12 weeks. Epididymal adipose tissue was obtained and subjected RT-PCR for expression of TNFα. High fat feeding increased TNFα mRNA (Figure 2A) in adipose tissue. Higher expression of TNFα was observed in epididymal and mesenteric depots compared to the subcutaneous depot (Figure 2B), and in the stromal vascular faction compared to adipocytes (Figure 2C). Bars represent means and ± SEM. Superscript letters represent significant mean differences, P < 0.05.

First, we determined the expression of SLPI in adipose tissue after a high fat (HF) diet. Increased protein and mRNA expression of SLPI was observed in epididymal fat from mice on high fat diet compared to mice on control (LF) diet (Figure 1A and 1B) (P < 0.05). Next, to determine if there are differences in SLPI expression in different adipose depots, we examined SLPI expression in subcutaneous, epididymal and mesenteric depots (Figure 1C). Highest expression of SLPI expression was found in adipose tissue from the mesenteric depot (P < 0.05) than the epididymal and subcutaneous depots. Additionally, significantly higher expression was found in the stromal vascular fraction than adipocytes (Figure 1D), an indication that this fraction is responsible for most of the increase in SLPI expression in adipose tissue in high fat diet. The higher expression SLPI in visceral depots (mesenteric and epididymal) than subcutaneous depot agrees with the higher level of TNFα, a classic marker of inflammation, observed in the epididymal tissue of mice on high fat diet, visceral (mesenteric and subcutaneous) vs. subcutaneous depot and in stromal vascular cells vs. adipocytes and whole adipose tissue (Figures 2A, B and 2C).

### Regulation of SLPI expression in adipocytes by inflammatory stimuli and anti-inflammatory effect of SLPI

**Figure 3:**
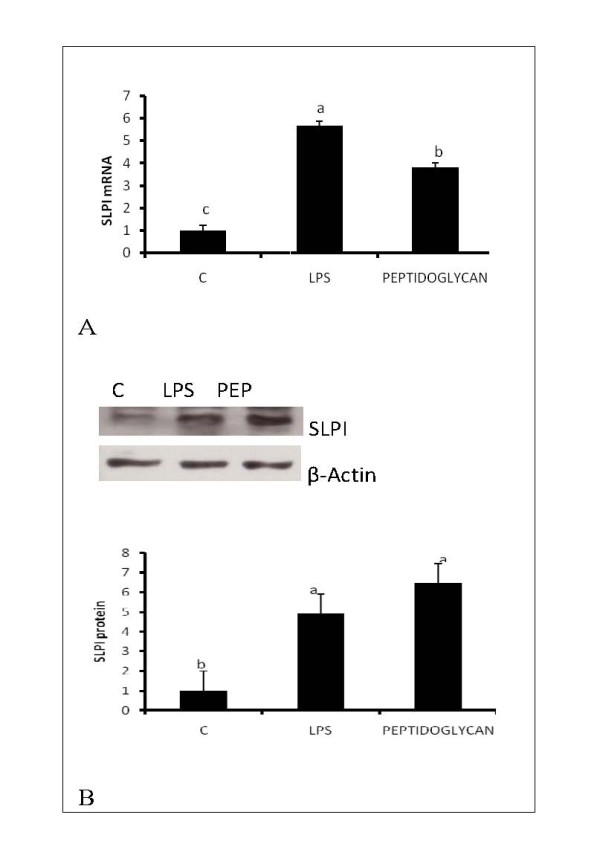
**Regulation of SLPI expression by LPS and peptidoglycan**. Differentiated 3T3-L1 adipocytes were either untreated (control, C) or treated with 100 ngml lipopolysaccharide (LPS) or peptidoglycan (PEP) (10 μg/ml) for 24 hours. SLPI mRNA was determined by RT-PCR and western blotting. Both LPS and peptidoglycan increased SLPI mRNA (Figure 3A) and protein (Figure 3B). Bars represent means and ± SEM of 4 different replicates. Superscript letters represent significant mean differences, P < 0.05.

**Figure 4:**
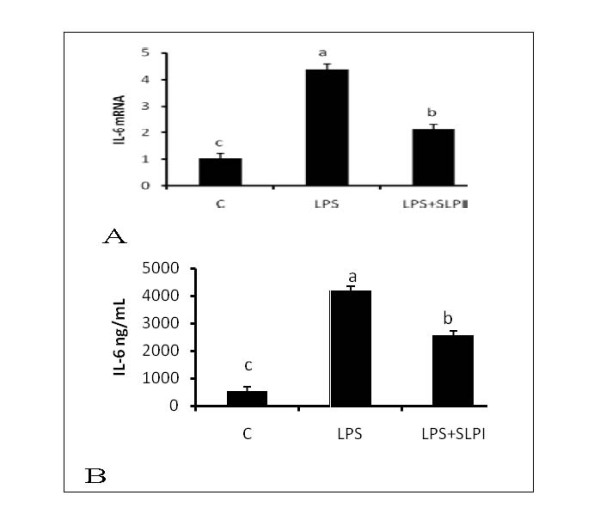
**Anti-inflammatory effects of SLPI in adipocytes**. Adipocytes were pretreated with 10 ng/ml recombinant SLPI for 2 hours and then treated with LPS (100 ng/ml) for 24 hours. Pretreatment with SLPI attenuates induction of IL6 mRNA (Figure 4A) and protein (Figure 4B). Bars represent means and ± SEM of 4 different replicates. Superscript letters represent significant mean differences, P < 0.05.

Adipocytes express both TLR2 and TLR4 and the expression of these receptors is upregulated in obesity. Therefore, we examined whether treatment of adipocytes with peptidoglycan and LPS, ligands for TLR2 and TLR4, could alter the expression of SLPI. Both peptidoglycan and LPS (Figures 3A and 3B) upregulated expression of SLPI (P < 0.05), suggesting that activation of these receptors in vivo could play a major part in the regulation of SLPI in adipose tissue. To determine whether SLPI exerts an anti-inflammatory role in adipocytes, 3T3-L1 adipocytes were pretreated for 2 hours with SLPI (10 ng/ml) and then with LPS (100 ng/ml) for 24 hours. Pretreatment of adipocytes with SLPI (Figures 4A and 4B) suppressed IL6 mRNA expression and protein secretion (P < 0.05). Therefore, SLPI may be an important protein that is induced in adipose tissue during obesity to dampen the inflammatory tone.

### SLPI stabilized IKBα expression in LPS treated adipocytes

**Figure 5:**
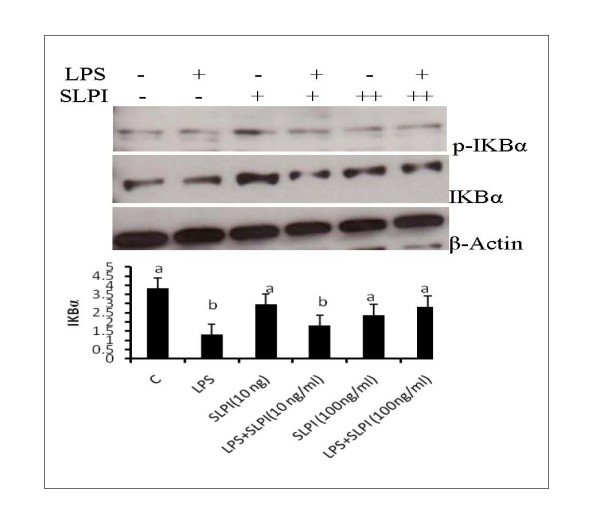
**SLPI stabilizes IKB α expression level**. Adipocytes were pretreated with 10 or 100 ng/ml recombinant SLPI for 2 hours and then treated with LPS (100 ng/ml) for 24 hours. Cell lysates were analyzed for the expression of phospho-IKB α and IKB α. Pretreatment with SLPI prevents the reduction of IKB α by LPS treatment (Figures 5A and B.) Bars represent means and ± SEM of 4 different replicates. Superscript letters represent significant mean differences, P < 0.05.

Due to the importance of IKBα as a negative regulator of TLR signaling, we investigated the effect of SLPI on the abundance of this protein. Pretreatment with SLPI resulted in significant stabilization of IKBα (Figures 5A and 5B), suggesting that stabilization of IKBα remains a possible mechanism by which SLPI counteracts inflammation in adipocytes.

## Discussion

Inflammation plays a major role in obesity-induced insulin resistance by the release of multiple inflammatory cytokines that oppose insulin signaling [14]. Although the endogenous mechanisms that trigger adipose tissue inflammation are not very clear, there is evidence that innate pattern recognition receptors such as TLR2 and 4 play key roles in this process [12,13]. These innate immune receptors are highly expressed in adipocytes and many functional assays have shown that their activation evokes inflammatory responses that are accompanied by increased expression of many inflammatory mediators (IL6, TNFα, MCP-1) [15,16]. However, mechanisms that lead to resolution of inflammation in adipose tissue are less well studied; despite the well established paradigm that initiation of inflammatory response is often accompanied by concurrent activation of feedback mechanisms that act to suppress inflammatory response [17-19]. In support of the presence of this mechanism in adipocytes, activation of NFκB and MAPK pathways in adipocytes by LPS is transient and rapidly returns to basal over time [20]. Although several mechanisms are behind the feedback mechanism of inflammation resolution [17-19], SLPI is recognized as a potent anti-inflammatory protein that is induced to suppress tissue inflammation [21]. Therefore, the increase in SLPI expression in adipose tissue in diet-induced obesity suggests that SLPI may play a role to antagonize inflammation in adipose tissue. The higher expression of SLPI in the stromal vascular fraction correlates well with the elevated expression of TNFα. This suggests that SLPI expression is induced in proportion to the degree of inflammation and agrees with a role for SLPI in dampening the inflammatory state. It also indicates that immune cells such as macrophages, which make up the bulk of the stromal vascular fraction may be the major source of adipose tissue SLPI. Therefore, counter-regulatory mechanisms exist in adipose tissue to suppress inflammation and SLPI may be part of these mechanisms. Although SLPI is highly expressed in mucosal surfaces [22,23], detection of its expression in adipose tissue indicates that it could play a key role in the resolution of inflammation in adipose tissue as well. Indeed, pretreatment of adipocytes with SLPI leads to downregulation of LPS induced IL-6 gene expression and protein secretion, confirming a functional role for SLPI in inflammation resolution in adipocytes. The anti-inflammatory action of SLPI may involve stabilization of IKBα abundance. Activation of TLR2 and 4 increased expression of SLPI in macrophages [10,24] and in adipocytes as confirmed in this study. Therefore, because TLR2 and TLR4 are activated in adipose tissue in obesity [12,13], the induction of SLPI in adipose tissue during obesity may be influenced by the activation state of the TLRs. Higher expression of SLPI in the visceral depots (mesenteric and epididymal) than the subcutaneous correlates with greater inflammation in the visceral depots than the subcutaneous depot. Elevated SLPI in the visceral depots could be part of the endogenous anti-inflammatory response to counter localized inflammation in these depots. Because visceral adiposity is linked to insulin resistance, induction of SLPI locally in adipose may also play a role in the prevention of inflammation-induced insulin resistance. In summary, we have demonstrated that obesity is accompanied by increased expression of SLPI in adipose tissue where it may act to suppress local inflammation.

## Abbreviations

ELISA: Enzyme linked immunoabsorbent assay; IKBα: Inhibitor of kappa B; IL6: interleukin 6; LPS: lipopolysaccharide; MCP: Monocyte chemoattracttant factor; NFκB: Nuclear factor kappa B; PEP: Peptidoglycan; SLPI: secretory leucocyte protease inhibitor; TLR: Toll-like receptors; TNF: Tumor necrosis factor.

## Competing interests

The authors declare that they have no competing interests.

## Authors' contributions

KMA conceived the original research idea. VJA assisted in the conduct of the experiments. KKB designed and supervised the in vivo mouse study. All authors read and approved the final manuscript.
